# Regionalization of Chinese Material Medical Quality Based on Maximum Entropy Model: A case study of *Atractylodes lancea*

**DOI:** 10.1038/srep42417

**Published:** 2017-02-16

**Authors:** Zhu Shoudong, Peng Huasheng, Guo Lanping, Xu Tongren, Zhang Yan, Chen Meilan, Hao Qingxiu, Kang Liping, Huang Luqi

**Affiliations:** 1 State Key Laboratory of Dao-di Herbs, National Resource Center for Chinese Materia Medica, China Academy of Chinese Medical Sciences, Beijing, China; 2 Anhui University of Chinese Medicine, School of pharmacy, Anhui, China; 3 State Key Laboratory of Earth Surface Processes and Resource Ecology, Faculty of Geographical Science, Beijing, Normal University, Beijing, China

## Abstract

*Atractylodes* is an East-Asiatic endemic genera that distributed in China, Japan and Russian Far Eastern. As an important resource of medicinal plant, *atractylodes* has long been used as herbal medicine. To example the significant features in its trueborn quality and geographical distribution, we explored the relationships between medicine quality and habitat suitability in two classifications–lower atractylodin content than the standard of *Chinese Pharmacopoei*a (2010) and the other has higher content. We found that the atractylodin content is negatively related to the habitat suitability for *atractylodes* with lower atractylodin, while the atractylodin content is positively related to the habitat suitability for those with higher atractylodin. By analyzing the distribution of *atractylodes*with lower atractylodin content than the standard of *Pharmacopeia*, we discovered that the main ecological factors that could inhibit the accumulation of atractylodin were soil type (39.7%), soil clay content (26.7%), mean temperature in December (22.3%), Cation-exchange capacity (6%), etc. And these ecological factors promoted the accumulation of atractylodin for the *atractylodes* with higher atractylodin. By integrating the two classifications, we finally predicted the distribution of atractylodin content in China.Our results realized the query of *atractylodes* quality in arbitrary coordinates, and satisfied the actually cultivation demands of “Planting area based on atractylodin quality”.

Regionalization, which means the division of regions, is a way that people extract the characteristic information of space and then classify and merge regions according to certain purposes in understanding the natural and social environment^1^,^2^,^3^,^4^,^5^. The regionalization of Chinese material medical resources was to study the Chinese material medical resources and their spatial variance in region system and regionalize the herbal medicine resources according to the similarity and variation of ecological characteristics^6^,^7^. Regionalization of Chinese herbal medicine has been studied since the 1990s, and the data came from the results of the Third National Census of Chinese material medicine resource. Great achievements in traditional Chinese medicine regionalization have been gained in the latest 20 years. But there still exists problems, for instance, the representativeness of sample is not enough meanwhile the accuracy and extent of ecology factor data is low, which resulting in the process of regionalization that always been affected by human’s subjective factors. At the same time, in the Chinese material medical planting industry, there often exist the problem that although the index component of Chinese material medical content conform to the standard of Pharmacopeia, they are not as good as the original region of traditional Chinese medicine, even some of them cannot reach to the standard of Pharmacopedia. Exploring the relationship between ecological factors of Chinese herbal medicine production areas and medical quality, optimizing the planting areas of traditional Chinese medicine, solving the practical problems during the process of Chinese traditional medicine production, and improving the reliability and practicability of the traditional Chinese medicine regionalization are the aims of the Chinese material medical quality regionalization.

Maximum Entropy Model is a species distribution model based on machine learning and the basics of ecology. It contains many advantages such that the different types of ecological factors are compatible, and in the progress of calculation, the effects of human’s subjective factors are eliminated and the model solely relies on the results of objective sampling. The model is widely used in studies of the coverage of animals and plants in terrestrial and marine ecosystems for its high accuracy^8^,^9^,^10^,^11^,^12^. The distribution and quality of the traditional Chinese medicine can be affected by natural environment. Habitat suitability is used to describe the quantitative index of relationship between ecological environment and the distribution of medical materials. We can establish the quantitative relationships between the ecological environment and the quality of Chinese traditional medicine by means of coupling correlation analysis, principle components analysis and clustering analysis.

As an important resource of medicinal plant, *atractylodes* is collected in national pharmacopoeia by China, Japan and Korea. *Atractylodes* has long been used as herbal medicine, and has significant features in its trueborn quality and geographical distribution^13^,^14^,^15^,^16^,^17^,^18^. In this research, we used *atractylodes* as a case to study the regionalization method of Chinese material medical quality. Specifically, based on the complete sample data and use the habitat suitability as the linkage, we studied the relationships between the quality of medical materials and ecological environment by the Maximum Entropy Model and other statistical analysis techniques. We also used the similarity of environment to grade the quality of medical materials and at last we achieved a map of regionalization of Chinese material medical quality.

The relationship between biology and environment is a core issue in ecology. The distributional suitability of organisms in the environment can be evaluated by species distribution model. The species distribution (it is also called habitat suitability model or environment niche model) covers many different models. It mainly relies on species distribution data which is already known to us and a series of environment variables to explore the species niche and its potential distribution. According to the data of model needed and its own principle, it can be divided into three different kinds, Group discriminant model, Frame model and Mechanism model^19^,^20^.

Entropy is an elementary concept in information theory. Shannon put forward the concept of entropy in an article of information theory domain in 1948 that entropy is used to describe the disorder of object, which is positively related to the degree of entropy, and the degree of uncertainness about object is called entropy^21^. Principle of maximum entropy (PME) adopts the principle to treat all the known and unknown objects: PME admits the known things while it does not make hypotheses for unknown things, which means that it keeps the uncertainness of object and lower the crisis to satisfy all known information. Under those conditions, attaining a probability distribution with less subjective prejudice, which is a most reasonable distribution^22^. The basic idea of PME is that offering a certain training sample and choosing a model according with the training sample. Maximum Entropy Model should choose a probability distribution conform to the discovery, and model gives a uniform probability distribution to other situations. The theory of PME is expressed in the bio ecology that one species would spreads and extends to the greatest extent possiblilities without any constraint condition, and close to the uniform probability distribution^23^,^24^. Steven Phillips and other people used the Java language to compile Maxent software on the basis of Maximum Entropy^10^.

In the software of Maxent, define *π* as a probability distribution of finite set *X*. For each *x* in the finite set *X, π*(*x*) as a probability distribution of this point and it must be non-negative. All *π*(*x*) are sum up to 1. What we estimated is also a probability distribution, define it as 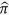. So we can get a formula to work out the entropy is 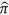.


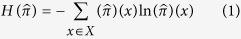


In the formula of eq. (1), ln is the natural logarithm. By using the method of Lagrange Multiplier Method, we can work out the probability distribution when the entropy is maximal.

## Methods

### Materials and Methods

**Figure 1:**
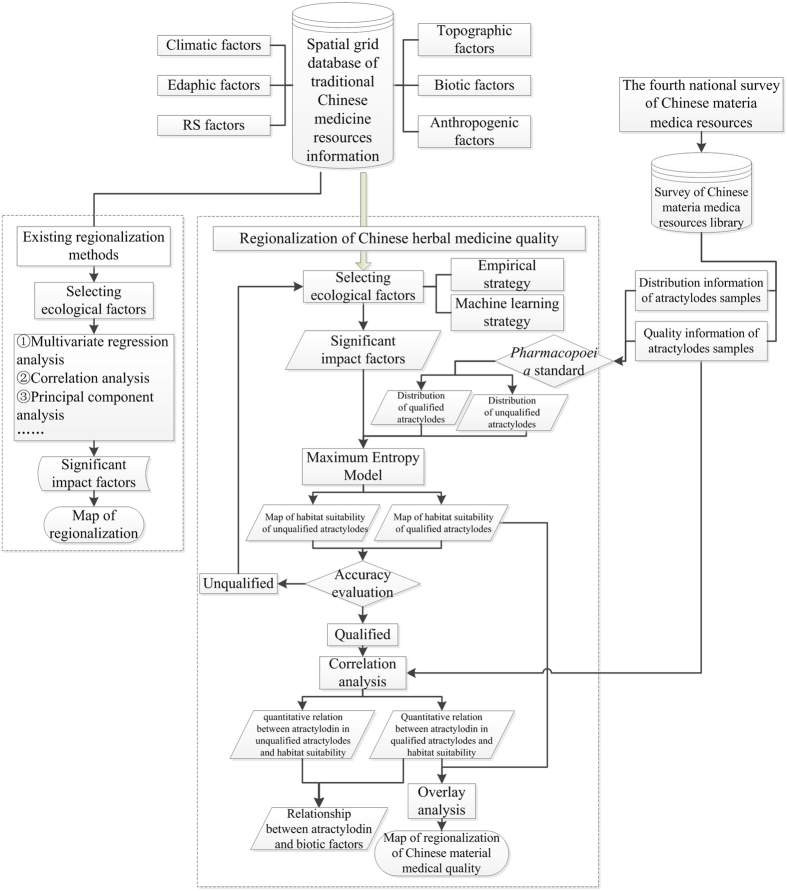
Map of technical route. Generated using the Visio version 14 software (Microsoft Inc., Redmond, WA, USA. URL: http://www.microsoft.com/).

*Atractylodes* has a long history of application and significant features in its trueborn quality, and was selected in this research. *Atractylodes* was collected from 20 sampling sites all over the nation and at each experimental site we sampled 4–5 duplicated samples. After a unified and standardized test, we can gain the average content data of atractylodin in each sampling site. According to the stipulation of atractylodin (C_13_H_10_O) content (content of atractylodin in the samples of oven dried *atractylodes* should not be less than 0.3%.) from the standard of *Chinese Pharmacopeia*^25^, sampling sites were divided into two parts, one is atractylodin with lowercontent than the standard of *Pharmacopeia*, and the other is atractylodin with higher content than the standard of *Pharmacopeia*. After that, we used maximum entropy model to calculate the habitat suitability of *atractylodes* separately to obtain the particular characteristic of medicinal material in habitat which atractylodin is higher than the standard of *Pharmacopeia* and atractylodin is lower than the standard of *Pharmacopeia*. Finally, by analyzing the promotive and inhibitive effect of habitat condition on accumulation of atractylodin, we obtained the quality regionalization map of *atractylodes* in national scale, and revealed the connections between the quality of medicinal material and habitat condition. The map of technical route was shown in Fig. 1.

### Experimental data

#### Data of ecological factors

Meteorological data came from the dataset of standard climatological annual and monthly value, which is obtained from 722 Chinese ground meteorological observation stations from 1950 to 2000, and the dataset of climatological annual, monthly and daily value, which gainsfrom 752 Chinese ground meteorological observation stations and automated stations since 1951^26^. By scientific analysis and calculation, we obtained the meteorological factor data for this study.

The data of soil type is arranged according to the 1:1000, 000 *Soil Map of China* (1995) offered by the second national survey of land^27^. This data can provide model input parameters for modeler. It can also be used to studying eco-agricultural division, food security, climate change and so on in agricultural perspective.

Terrain data includes elevation, slope and aspect^28^.

The data of vegetation type is arranged according to the sub-type vegetation data that come from to the 1:1000, 000 *Vegetation Map* of China published by Institute of botany, Chinese Academy of Sciences^29^.

Comprehensive meteorological indices come from warmth index and coldness index in Kira index^30^, and humidity index that modified from Kira index by Xu Wenduo^31^.

#### The distribution information of *atractylodes*

The geographic range of *atractylodes* was further precisely checked by continuous field investigation from July to November in 2011. In each sampling site, altimeter was utilized to determine elevation, GPS was used to measure longitude and latitude, and the investigation of plant population was carried out. According to The Plant List (http://theplantlist.org) *Atractylodes lanceae* was regarded the species name in this research. 2–5 samples were obtained from each of the 20 sample sites.

#### The chemical composition content of *atractylodes*

**Figure 2:**
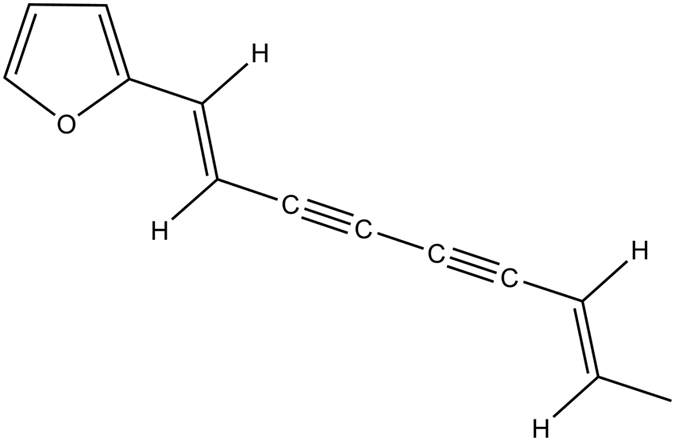
Chemical structure of atractylodin. Generated using the ChemBioDraw Ultra 12.0 software (PerkinElmer Inc., Wellesley, MA, USA. URL: http://www.cambridgesoft.com/).

Gas Chromatography-Mass Spectrometer was used in chromatographic analysis of the volatile oil component standard substances in this study to get the standard curve of standard substances. The method is verified to be stable after repetitive experiments. And then, chromatographic analysis was operated with samples from the field. 4–5 replicate samples were obtained from each sample site. Comparisons of chromatogram between samples and standard substances were performed and finally 4 kinds of chemical composition content value of *atractylodes* were obtained (see Supplementary material 1). The chemical structure of atractylodin was shown in Fig. 2.

### Selection of ecological factors

The correlation coefficient between each ecological factor and atractylodin content was calculated by relevance strategy, and the data in descending order was sorted according to absolute value of correlation coefficient. We found that temperature plays a key role in the accumulation of *atractylodes* essential oil component and precipitation has a significant influence on the survival of *atractylodes* by experience strategy and the living habits of *atractylodes*. With the consideration that climatological monthly value is better than annual value in reflecting the requirement of climate conditions by species. These ecological factors whose correlation coefficient with atractylodin is more than 0.2 were selected in this study, as shown in Table 1.

**Figure 3:**
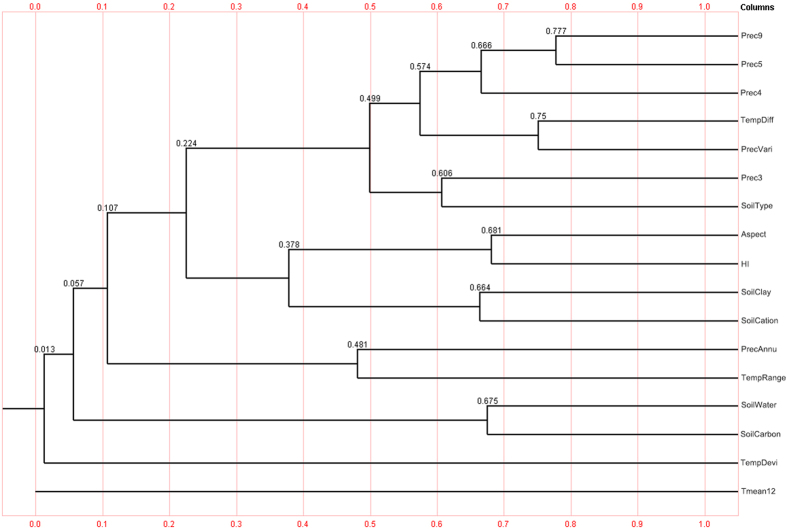
The correlation coefficient tree diagram of 17 ecological factors. Generated using the BioSim2 version 2 software (Information Technology Department of Norwich University, Las Vegas, NE, USA).

The correlation coefficient of these factors and *atractylodes* contentwere calculated. We found that the mean precipitation in March and November, April and October whose correlation coefficient are more than 0.8, thus the mean precipitation in November and October were deleted. Finally, the correlation coefficient tree diagram of 17 ecological factors was obtained as shown in Fig. 3.

### Maximum Entropy Model Calculation

Maxent model needs two sets of data to operate: (1) the distribution data of *atractylodes* in the form of latitude and longitude; (2) the ecological factor data of *atractylodes* in the study area. The operation processes of Maxtent model are as follows: first of all, obtain the distribution data and ecological factors of *atractylodes* in the study area; secondly, operate the model to choose suitable predictable ecological factors for the growth of *atractylodes*; then, establish predictable model and predict the potential geographical distribution for target species; finally, work out a Fig and evaluate the predictable accuracy of the model. Maxent assumes a uniform distribution of ecological factors at first and then proceeds iteration; the importance of every ecological factor in the formula is adjusted constantly during the process of iteration, which is called Training Gain. Maximizing the average probability of each grid value which is got in the end, thus the predictable distribution Fig is obtained in the Maxent. Output value is continuous in a certain range, and each grid has a value to show the suitable degree of *atractylodes* to the ecological factor in the grid. The value in the grid net is a cumulative probability, and the bigger the grid value, the more possible the species suitable degree.

The parameter is set as follows when using Maxent 3.3.3 software: Convergence threshold is set at 0.00005, Maximum iterations is set at 1000000, other parameters are set to the default. 15% of parameters were selected as Random test percentage randomly in Samples, the rest data is set as the training data. The distribution data of *atractylodes* with higher and lower atractylodin content than the standard of *Pharmacopedia* was selected respectively to calculate the habitat suitability of the *atractylodes* in the national scale.

### The Accuracy Evaluation of Model Calculation

**Figure 4:**
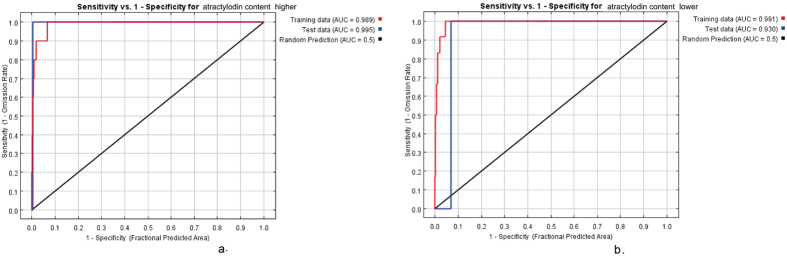
Receiver operating characteristic curve, (**a**) atractylodin content is higher the standard of *Pharmacopeia*, (**b**) atractylodin content is lower than the standard of *Pharmacopeia*. Generated using the Maxent version 3.3.3 k software (AT&T Labs–Research, Florham Park, NJ, USA. URL: http://www.cs.princeton.edu/~schapire/maxent/).

Figure 4 showed the curve of the receiver operating characteristic (ROC) which is based on the training set and testing set. The 1-Specificity of the abscissa is not real debugging but is defined by predicted area. And that means the fractional predicted area can receive the biggest AUC is less than 1. Abscissa 1-specificity is false positive rate, vertical ordinate 1-Omission is true positive rate, blue line is the ROC curve of testing set, and the area which is below the ROC curve is called AUC(Area under roc Curve) value. In this study, the AUC value of testing data is 0.995 when the habitat suitability of *atractylodes* with lower atractylodin content than the standard of *Pharmacopoeia*, and the AUC value of testing data is 0.930 when the habitat suitability of *atractylodes* with higher atractylodin content than the standard of *Pharmacopoeia*. It indicates that the accuracy of *atractylodes* habitat suitability is high on the basis of two groups of training set.

## Results

### The results of atrctylodes habitat suitability distribution

#### The habitat suitability distribution of *atractylodes* with lower atractylodin content than the standard of *Pharmacopoeia*

According to the result which was calculated by Maximum Entropy Model, the contribution ratios of various ecological factors to the habitat suitability of *atractylodes* with lower atractylodin content than the standard of *Pharmacopoeia* were obtained. As shown in Table 2, under the circumstance that the correlation of various ecological factor is not significant, the types of soil (39.7%), the clay content of soil (26.7%), the mean temperature in December (22.3%), the cation exchange capacity of the soil (6%) have significant influences on the distribution of *atractylodes* with lower atractylodin content than the standard of *Pharmacopoeia*.

**Figure 5:**
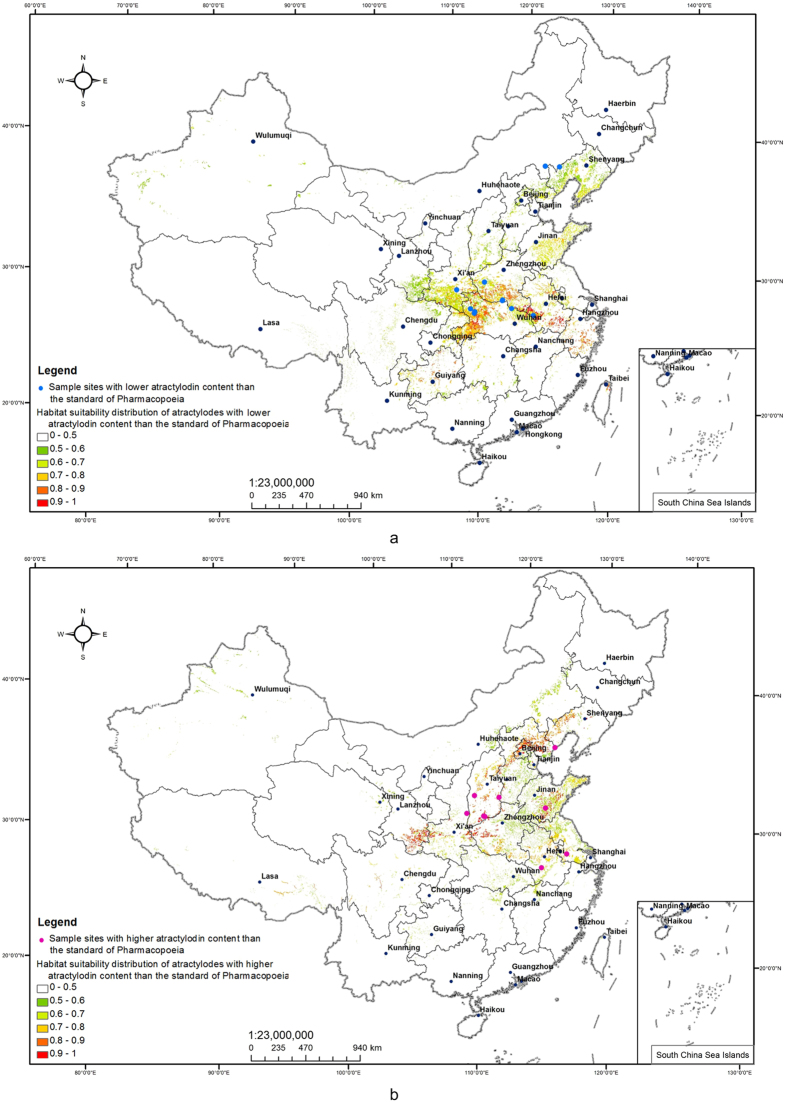
(**a**) The habitat suitability distribution of *atractylodes* with lower atractylodin content than the standard of *Pharmacopoeia*. (**b**) The habitat suitability distribution of *atractylodes* with higher atractylodin content than the standard of *Pharmacopoeia.* Generated using the ArcMap version 10.0 software (ESRI Inc., California, USA. URL: http://www.esri.com/).

At the same time, the habitat suitability distribution of *atractylodes* with lower atractylodin content than the standard of *Pharmacopoeia* was calculated by model, which was shown in Fig. 5a. The range of 0~1 was used to show the suitable situation of environment for the growth of *atractylodes*, the bigger the value, the better the adaptability. The central and southern of Zhejiang, southern of Anhui, eastern of Hubei, southern of Henan, southern of Shaanxi, middle of Shandong, southern of Liaoning are the most suitable areas for the growth of *atractylodes* with lower atractylodin content than the standard of *Pharmacopoeia*, which means that *atractylodes* grown in these areas had a lower content of atractylodin than the standard of *Pharmacopoeia*.

#### The habitat suitability distribution of *atractylodes* with higher atractylodin content than the standard of *Pharmacopoeia*

According to the result which was calculated by Maximum Entropy Model, the contribution ratios of various ecological factors to the habitat suitability of *atractylodes* with higher atractylodin content than the standard of *Pharmacopoeia* were obtained. As shown in Table 3, under the circumstance that the correlation of various ecological factor is not significant, the types of soil (50.0%), the clay content of soil (34.8%), the mean temperature of 12 month (10.0%), the aspect (1.8%)and available water content of soil (1.6%) have significant influences on the distribution of *atractylodes* with higher atractylodin content than the standard of *Pharmacopoeia*.

At the same time, the habitat suitability distribution of *atractylodes* with higher atractylodin content than the standard of *Pharmacopoeia* was calculated by model, which was shown in Fig. 5b. The middle of Anhui, middle and eastern of Jiangxi, eastern of Henan, southern and eastern of Gansu, southern of Shandong, middle and southern of Shanxi, northern of Hebei and Beijing, eastern of Liaoning are the most suitable areas for the growth of *atractylodes* with higher atractylodin content than the standard of *Pharmacopoei*a, which means that *atractylodes* growing on those these areas of which the content of atractylodin is higher than the standard of *Pharmacopoeia*. The habitat suitability in every sampling site shows in Table 4.

### Correlation analysis results of atractylodin and habitat suitability

#### *Atractylodes* with lower atractylodin content than the standard of *Pharmacopoeia*

**Figure 6:**
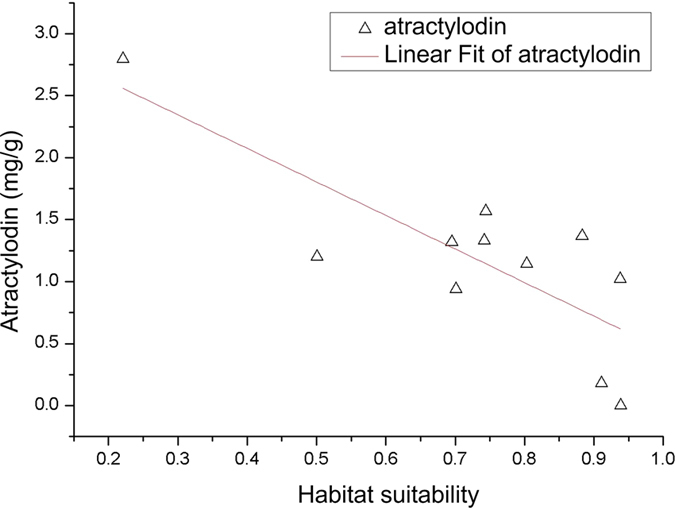
Relationships of atractylodin content and the habitat suitability of *atractylodes* with lower atractylodin content than the standard of *Pharmacopoeia.* Generated using the OriginPro version 8.0 software (OriginLab Corp., Northampton, MA, USA. URL: http://www.OriginLab.com/).

*Atractylodes* with lower atractylodin content (y) than the standardof *Pharmacopoeia* and habitat suitability (x) were used to do correlation analysis in 11 sampling sites. The results show in Fig. 6, and the linear relation is:


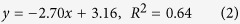


By F test (P = 0.003, F = 15.81), habitat suitability is negatively correlated with the content of atractylodin. Combined with Table 2, the main ecological factors such as soil type, soil clay content, the mean temperature in December, cation exchange capacity of the soil and aspect restrained the accumulation of atractylodin content.

#### *Atractylodes* with higher atractylodin content than the standard of Pharmacopoeia

**Figure 7:**
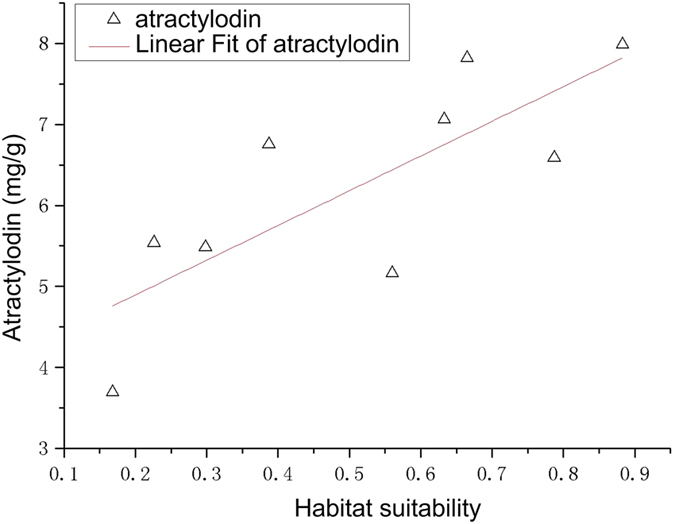
Relationships of atractylodin content and the habitat suitability of *atractylodes* with higher atractylodin content than the standard of *Pharmacopoeia.* Generated using the OriginPro version 8.0 software (OriginLab Corp., Northampton, MA, USA. URL: http://www.OriginLab.com/).

*Atractylodes* with higher atractylodin content (y) than the standard of *Pharmacopoeia* and habitat suitability (x) were used to do correlation analysis in 9 sampling sites. The results were shown in Fig. 7, and the linear relationship is:


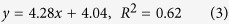


By F test (P = 0.012, F = 11.29), habitat suitability is positively correlated with atractylodin content. Combined with Table 3, the main ecological factors such as soil type, soil clay content, the mean temperature in December, aspect and available water content of soil promoted the accumulation of atractylodin content.

### The influence of each significant ecological factor to the accumulation of atractylodin content

We can learn from the correlation analysis results that soil types, soil clay content, the mean temperature of 12 month and aspect have the same effect on the accumulation of atractylodin content, and the ecological factors that influenced the atractylodin content accumulation of the two kinds of *atractylodes* were studied separately.

#### Soil type

**Figure 8:**
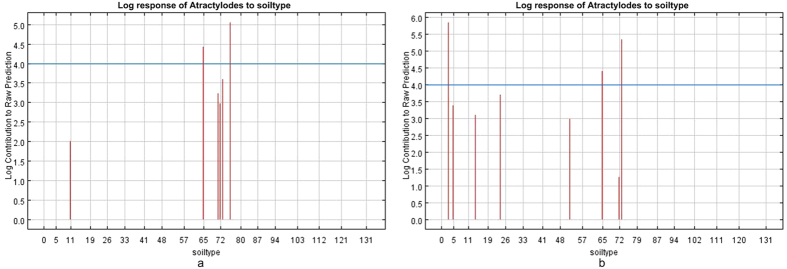
Response-function diagram of soil clay content to the habitat suitability of *atractylodes*. (**a**) *Atractylodes* with lower atractylodin content than the standard of *Pharmacopoeia*, (**b**) *Atractylodes* with higher atractylodin content than the standard of *Pharmacopoeia*. Generated using the Maxent version 3.3.3k software (AT&T Labs–Research, Florham Park, NJ, USA. URL: http://www.cs.princeton.edu/~schapire/maxent/).

According to the explanation of soil data comes from Traditional Chinese Medicine Resources Spatial Information Grid Database and combining with Fig. 8, *atractylodes* with lower atractylodin content than the standard of *Pharmacopoeia* was restrained in the environmental effects of unsaturated dystric regosols and saturated eutric planosols; while in the environmental effect of uncultivated land of urban and calcic luvisols, atractylosed with higher atractylodin content than the standard of *Pharmacopoeia* was promoted. At the same time, we can know that eutric planosols are important for the accumulation of atractylodin content, but also have universality.

The humus layer of dystric regosols was almost eroded, severe soil erosion and the content of organic materials are few were found after analyzing these kinds of soil, so this kind of soil restrained the accumulation of atractylodin. The content of the calcic luvisols is high, and surface permeable rate is fine, so the soil is always in the situation of continuous dry-wet alternation. Middle permeable rate is low, formed a natural water-resisting layer, which has a stimulative impact for the accumulation of atractylodin content. The particles of eutric planosols are of high-density, thus the permeable rate is low, and the soil shows different charactistics during the period of abundant precipitation and rare precipitation.

#### Soil clay content

**Figure 9:**
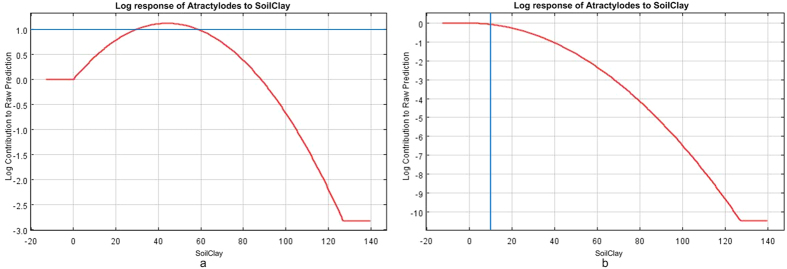
Response-function diagram of soil clay content to the habitat suitability of *atractylodes*. (**a**) *Atractylodes* with lower atractylodin content than the standard of *Pharmacopoeia*, (**b**) *Atractylodes* with higher atractylodin content than the standard of *Pharmacopoeia*. Generated using the Maxent version 3.3.3 k software (AT&T Labs–Research, Florham Park, NJ, USA. URL: http://www.cs.princeton.edu/~schapire/maxent/).

As shown in Fig. 9, *atractylodes* with lower atractylodin content than the standard of *Pharmacopoeia* was restrained when the content of clay soil ranges from 28% to 58%; and atractylodin content higher than the standard of *Pharmacopoeia* was promoted when the content of clay soil ranges from 0% to 10%. The speed of water penetration becomes slow and the water retention capacity is fine which were caused by high content of soil clay. Conversely, it restrained the accumulation of atractylodin content.

#### The mean temperature in December

**Figure 10:**
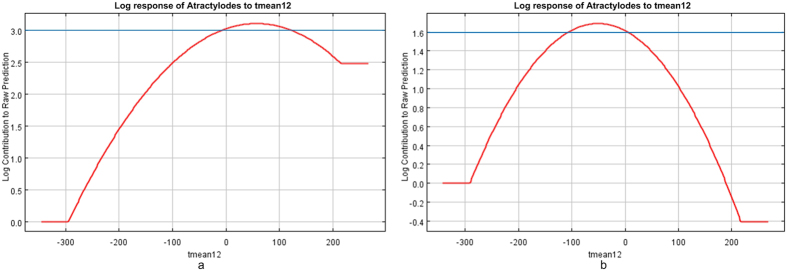
Response-function diagram of the mean temperature in December to the habitat suitability of *atractylodes*. (**a**) *Atractylodes* with lower atractylodin content than the standard of *Pharmacopoeia*, (**b**) *Atractylodes* with higher atractylodin content than the standard of *Pharmacopoeia*. Generated using the Maxent version 3.3.3 k software (AT&T Labs–Research, Florham Park, NJ, USA. URL: http://www.cs.princeton.edu/~schapire/maxent/).

As shown in Fig. 10, *atractylodes* with lower atractylodin content than the standard of *Pharmacopoeia* was restrained when the mean temperature in December ranges from 0 to 11 °C; and atractylodin content higher than the standard of *Pharmacopoeia* was promoted when the mean temperature in December temperature ranges from −10 to 0 °C. The result is coincide with the habitat characteristic of truebornmedicinal materials of *atractylodes* that the mean temperature ranges from −2 to −1 °C in coldest month at original place, which was summarized by Guo Lanping and Huang Luqi^32^,^33^.

#### Aspect

**Figure 11:**
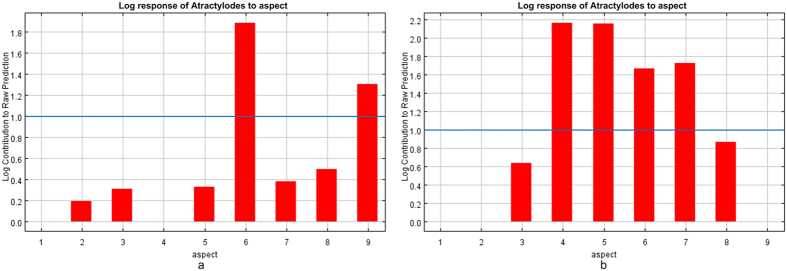
Response-function diagram of aspect to the habitat suitability of *atractylodes*. (**a**) *Atractylodes* with lower atractylodin content than the standard of *Pharmacopoeia*, (**b**) *Atractylodes* with higher atractylodin content than the standard of *Pharmacopoeia*. Generated using the Maxent version 3.3.3k software (AT&T Labs–Research, Florham Park, NJ, USA. URL: http://www.cs.princeton.edu/~schapire/maxent/).

The corresponding relation of aspect and azimuthal angle was shown in Fig. 11 according to Traditional Chinese medicine resources information spatial database. Atractylodin with lower content than the standard of *Pharmacopoeia* was restrainedwhen the aspect towards south (157.5°–202.5°, 6)and northwest (292.5°–337.5°, 9); atractylodin with higher content than the standard of *Pharmacopoeia* was promoted when the aspect towards east (67.5°–112.5°, 4), southeast (112.5°–157.5°, 5), southwest (202.5°–247.5°, 7)and south (157.5°–202.5°, 6). It is because the solar radiation in south area is more than other orientation and the solar radiation in north area is minimal in the mountain area. The extreme of too hot and too cold restrains the accumulation of atractylodin content. Comparatively, the solar radiation towards east, southeast and southwest is not the strongest, but the moderate. The result is coincided with the trueborn medicinal materials of *atractylodes* that atractyloses prefers to grow in a place where with low temperature and humidity than a place with strong light and high temperature, which was summarized by Sun Yuzhang and Guo Lanping^34^,^35^.

### The distribution of atractylodin content

#### The distribution of atractylodin content in national map

The calculation method of the predicted atractylodin is as follows. Equation 3 is used to calculate the predicted atractylodin contentin the pointwhen the habitat suitability higher than the standard of *Pharmacopoeia* is greater than or equal to the habitat suitability lower than the standard of *Pharmacopoeia*; eq. 2 is used to calculate the predicted atractylodin content in the point when the habitat suitability higher than the standard of *Pharmacopoeia* is less than the habitat suitability lower than the standard of *Pharmacopoeia*.

By analyzing *atractylodes* habitat suitability distribution, Correlation analysis results and focusing on the habitat suitability map of atratylodes with lower atractylodin content than the standard of *Pharmacopeia*, we found that the southwestern of Anhui, southern of Henan, western of Hubei and southern of Zhejiang were more suitable for the growth of *atractylodes*, but atractylodin content was strongly restrained by the environment and could hardly reach to the standard of *Pharmacopeia*.

**Figure 12:**
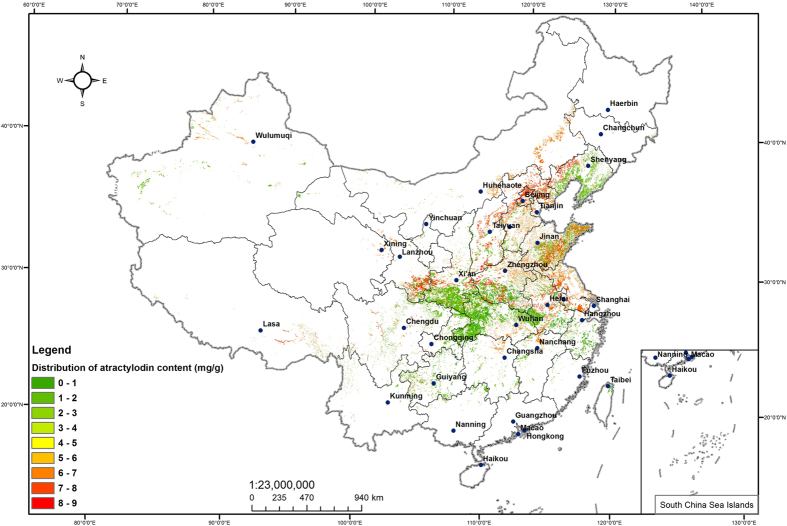
The distribution of atractylodin content. Generated using the ArcMap version 10.0 software (ESRI Inc., California, USA. URL: http://www.esri.com/).

The middle-northern of Hebei, western of Liaoning, western of Hunan, southern of Shanxi, southeastern of Shandong and southern of Gansu suit for the growth of *atractylodes* after analyzing the habitat suitability map of *atractylodes* with higher atractylodin content than the standard of *Pharmacopeia*. And at the same time, atractylodin content higher than the standard of *Pharmacopeia* was strongly promoted by the environment. The distribution of atractylodin content was shown in Fig. 12.

#### Verification of atractylodin content distribution

The distribution and component of *atractylodes* data was collected by researchers Guo Lanping^26^ and Zhang Yan^36^ in our research group. The measured and predicted atractylodin content were shown in Table 5.

Verification by relative error The relative error is the rate between the measurement of absolute error and the true measured value. The data of *atractylodes* in 10 verification points were plugged into eq. 3 to examine the predicted results of atractylodin. Finally, we obtain the relative error of predicted atractylodin that is 28.00%.


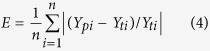


*E* is final relative error of predicted atractylodin; *n* is the number of verification point; *Y*_*p*_ means the content of predicted atractylodin, and *Y*_*t*_ means the content of measured atractylodin.

Verification by root-mean-square error Root-mean-square error is used to measure the error between observed and true values. The data of *atractylodes* in 10 verification points were plugged into eq. 4 to examine the predicted results of atractylodin. Finally, we obtain the relative error of predicted atractylodin that is 1.06 mg/g.


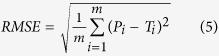


in eq. 5, *P*_i_ is predicted value in the *i*^th^ verification points, *T*_i_ is measured value in the *i*^th^ verification points.

## Discussion

### Comparison of study results with others

Our studies were coincident with the resultsof previous studies in the habitat suitability of *atractylodes*.

### Analysis of the result of quality regionalization

From the correlation analysis of the quality of *atractylodes* and habitat suitability, the results are as follows:

Evaluation on the quality of *atractylodes* was based on the stipulation of atractylodin (C_13_H_10_O) content (content of atractylodin in the samples of oven dried *atractylodes* should not be less than 0.3%) according to the 2010 edition of the *Pharmacopeia*. For *atractylodes* with lower atractylodin content than the standard of *Pharmacopeia*, the content of atractylodin (y) is negatively correlated with the habitat suitability (y = −2.70x + 3.16, *R*^2^ = 0.64). While for those with higher atractylodin content than the standard of *Pharmacopeia*, the content of atractylodin (y) is positively correlated with the habitat suitability (y = 4.28x + 4.04, *R*^2^ = 0.62).

By analyzing the distribution of *atractylodes* with lower atractylodin content than the standard of *Pharmacopeia*, we discovered that the factors (with their contribution ratio in brackets) which could constrain the accumulation of atractylodin content are soil type (39.7%), soil clay content (26.7%), the mean temperature in December (22.3%), Cation-exchange capacity (6%), etc. By analyzing the distribution of *atractylodes* with higher atractylodin content than the standard of *Pharmacopeia*, we discovered that the factors which could constrain the accumulation of atractylodin content are soil type (50%), soil clay content (34.8%), the mean temperature in December (10%), etc. The detailed factors are as follows:

Soil type: atractylodin content was restrained in the environmental effects of unsaturated dystric regosols and saturated eutric planosols; while atractylodin content was promoted in the environmental effect of uncultivated land of urban and calcic luvisols.

Soil clay content: atractylodin content was restrained when the content of clay soil ranges from 28% to 58%; and areactylodin content was promoted when the content of clay soil ranges from 0% to 10%.

The mean temperature in December: atractylodin content was restrained when the mean temperature in December ranges from 0 to 11 °C; and atractylodin content was promoted when the mean temperature in December temperature ranges from −10 to 0 °C.

Aspect: atractylodin content was restrained in condition that the aspect towards south and northwest; and atractylodin content was promoted in the condition that the aspect towards east, southeast, and southwest.

### Result of quality regionalization of *atractylodes* in national scale

In this study, the query of *atractylodes* quality in arbitrary coordinate was realized, and the actually cultivation demands of “planting area based on atractylodin quality” was satisfied. Combined with the cultivation industry of traditional Chinese medicine, the planting area where the distribution of atractylodin content is high, and use scientific field management techniques to conduct ecological factors in the field can help improve the economic profit of planting *atractylodes*.

## Additional Information

**How to cite this article**: Shoudong, Z. *et al*. Regionalization of Chinese Material Medical Quality Based on Maximum Entropy Model: A case study of *Atractylodes lancea. Sci. Rep.* **7**, 42417; doi: 10.1038/srep42417 (2017).

**Publisher's note:** Springer Nature remains neutral with regard to jurisdictional claims in published maps and institutional affiliations.

## Supplementary Material

Supplementary Material 1

## Figures and Tables

**Table 1:** Significant impact factors list.

Sequence number	Ecological factors	Abbreviation	Correlation coefficient ofatractylodin content
1	Soil type(based on FAO-90)	SoilType	0.530
2	Soil clay content(0–30 cm topsoil)	SoilClay	0.519
3	Soil organic carbon content(0–30 cm topsoil)	SoilCarbon	0.465
4	The mean precipitation in May	Prec5	0.449
5	Cation exchange capacity of the soil(0–30 cm topsoil)	SoilCation	0.444
6	The mean precipitation in October	Prec10	0.441
7	Seasonal precipitation variation coefficient	PrecVari	0.377
8	Soil available water content level	SoilWater	0.351
9	The mean precipitation in April	Prec4	0.346
10	Aspect	Aspect	0.301
11	The mean precipitation in September	Prec9	0.295
12	Mean annual precipitation	PrecAnnu	0.289
13	Standard deviation of the seasonal changes in temperature	TempDevi	0.283
14	Humid index	HI	0.273
15	The range of mean annual temperature	TempRange	0.265
16	Monthly mean of temperature difference between day and night	TempDiff	0.255
17	The mean precipitation in March	Prec3	0.247
18	The mean precipitation in November	Prec11	0.244
19	The mean temperature in December	Tmean12	0.233

**Table 2:** The contribution ratios of various ecological factors to the habitat suitability of *atractylodes* with lower atractylodin content than the standard of *Pharmacopoeia.*

Sequence number	Ecological factors	Contribution (%)
1	Soil type	39.7
2	Soil clay content	26.7
3	The mean temperature in December	22.3
4	Cation exchange capacity of the soil	6.0
5	Aspect	2.3
6	Standard deviation of the seasonal changes in temperature	1.2
7	The mean precipitation in November	1.0
8	Soil organic carbon content	0.4
9	Monthly mean of temperature difference between day and night	0.2

**Table 3:** The distribution of various ecological factors to the habitat suitability of *atractylodes* with higher atractylodin content than the standard of *Pharmacopoeia.*

Sequence number	Variable name	Contribution (%)
1	Soil type	50.0
2	Soil clay content	34.8
3	The mean temperature in December	10.0
4	Aspect	1.8
5	Soil available water content level	1.6
6	Monthly mean of temperature difference between day and night	1.1
7	The mean precipitation in May	0.3
8	Standard deviation of the seasonal changes in temperature	0.1
9	The mean precipitation in November	0.1
10	Soil organic carbon conten	0.1

**Table 4:** The list of atractylodin content in sampling site.

Number	Location	Atractylodin(mg/g)	Habitat suitability
1	Jigongshan, Henan	0.178	0.911
2	Tongbai, Henan	1.567	0.744
3	Tantou, luanchan, Henan	0.940	0.701
4	Suizhou, Hubei	1.143	0.803
5	Guoduwan, Baokang, Hubei	1.319	0.695
6	Shennongjia forestry district, Hubei	1.022	0.938
7	Songluo, Shennongjia forestry district, Hubei	1.332	0.742
8	Jiezi, Zhenan, Shaanxi	1.367	0.883
9	Huangjia, Tongcheng, Anhui	7.062	0.633
10	Taipingfan, Huoshan, Anhui	0.000	0.939
11	Maoshan, Jiangsu	7.984	0.883
12	Qinhuangdao, Hebei	5.162	0.560
13	She xian, Handan, Hebei	6.756	0.387
14	Hongshan, Chifeng, Innermongolia	2.794	0.221
15	Daheishan, Chifeng, Innermongolia	1.198	0.501
16	Mengshan, Mengyin, Shandong	7.818	0.665
17	Jiaokou, Shanxi	6.588	0.787
18	Yuanqu, Shanxi	5.537	0.226
19	Yuanqu, Shanxi	5.482	0.298
20	Huanglong shan, Hancheng	3.693	0.168

**Table 5:** The information of *atractylodes* in verification point.

SequenceNumber	Location	Measured Atractylodin(mg/g)	Predicted atractylodin(mg/g)
1	Liyang, Jiangsu	3.5	4.689
2	Jurong, Jiangsu	3.4	1.963
3	Taiping, Huangshan, Anhui	3.4	4.785
4	Wangzhuang, Changan, Shaanxi	1.6	2.212
5	Huayin, Shaanxi	1.8	2.332
6	SongshanMountain, dengfendeg, Henan	6.7	5.267
7	Chongli, Hebei	5.6	7.257
8	Zhangshiyan, zanhuang, Hebei	2.3	2.406
9	Wangjiadian, Taipingfan, Huoshan	1.896	1.365
10	Guangde, Anhui	2.378	2.656

## References

[b1] Bailey R. G., Zoltai S. C. & Wiken E. B. Ecological regionalization in Canada andthe United States. Geoforum. 16, 265–275 (1985).

[b2] Margules C. R. & Pressey R. L. Systematic conservation planning. Nature. 405, 243–253 (2000). 10821285 10.1038/35012251

[b3] Margules C. R., Pressey R. L. & Williams P. H. Representing biodiversity: dataand procedures for identifying priority areas for conservation. J. Biosciences. 27, 309–326 (2002). 10.1007/BF02704962 12177531

[b4] Reyers B., Wessels K. J. & Van Jaarsveld A. S. An assessment of biodiversitysurrogacy options in the Limpopo Province of South Africa. Afr. Zool. 37, 185–195 (2002).

[b5] Ward T. J., Vanderklift M. A., Nicholls A. O. & Kenchington R. A. Selectingmarine reserves using habitats and species assemblages as surrogates forbiological diversity. Ecol. Appl. 9, 691–698 (1999).

[b6] Chinese Medical Company. Chinese Material Medical Regionalization. Beijing: Science Press (1995).

[b7] Zhu S. D. . 20 Years of Chinese Materia Medica Regionalization: from Single Herbal Regionalization to Regional Regionalization. Modern Chin. Med. 16, 91–95 (2014).

[b8] Antoine G. & Niklaus E. Z. Predictive habitat distribution models in ecology. Ecol. Model. 135, 147–186 (2000).

[b9] Steven J. P., Miroslav D. & Robert E. S. “A maximum entropy approach to species distribution modeling.” In Proc. the Twenty-First International Conference on Machine Learning, 655–662 (2004).

[b10] Steven J. P., Robert P. A. & Robert E. S. Maximum entropy modeling of species geographic distributions. Ecol. Model. 190, 231–259 (2006).

[b11] Jane E. . A statistical explanation of MaxEnt for ecologists. Divers. Distrib. 17, 43–57 (2011).

[b12] Elith J. . Novel methods improve prediction of species’ distributions from occurrence data. Ecography. 29, 129–151 (2006).

[b13] Guo L. P. . Comparing of Different Methods on Habitat Adaptive Division of Chinese Material Medica. China J. Chin. Materia Medica. 33, 718–724 (2008). 18590205

[b14] Guo L. P. . Key Influencing Factors on Essential Oil Component of Atractylodes Lancea and Study on its Division of Climate Adaptability. China J. Chin. Materia Medica. 32, 888–893 (2007). 17655137

[b15] Guo L. P., Huang L. Q., Yan H. & Jiang Y. X. Habitat Characteristics for the Growth of Atractylodes Lancea Based on GIS. China J. Chin. Materia Medica. 30, 565–569 (2005). 16011274

[b16] Zhang Y. . Effects of Different Microhabitas on Growth and Four Kinds of Volatile Oil Components of Atractylodes Lancea. China J. Chin. Materia Medica. 40, 4142–4148 (2015). 27071246

[b17] Takeda O., Miki E., Higuchi M. & Okada M. Historical Investigation on Quality Evaluation of Atractylodes lancea Rhizome (Cangzhu; Soujutsu) (II) : On the Japanese Herbal Literature. Jap. J. history pharm. 33, 24–28 (1998).

[b18] Jehyun L., Yunkyung K., Seonpyo H. & Chungsook K. Studies of taxonomic origins of Atractylodis Rhizoma Alba and Atractylodis Rhizoma. Kor. J. Oriental Medi. 8, 55–63 (2002).

[b19] Miguel B. A. & Antoine G. Five (or so) challenges for species distribution modelling. J. Biogeogr. 33, 1677–1688 (2006).

[b20] Peterson A. T. Predicting the geography of species’ invasions via ecological niche modeling. Q. Rev. Biol. 78, 419–433 (2003). 14737826 10.1086/378926

[b21] Shannon C. E. A mathematical theory of communication. Bell Syst. Tch. 27, 379–423 (1984).

[b22] Jaynes E. T. Fundamental Theories of Physics, Kluwer Academic Publisher (1990).

[b23] Jaynes E. T. Information theory and statistical mechanics. Phys. Rev. 108, 171–190 (1957).

[b24] Li M. Y., Ju Y. W., Kumar S. & Stohlgren T. J. Modeling potential habitat for alien species of Dreissena polymorpha in the Continental USA. Acta Ecol. Sin. 28, 4253–4258 (2008).

[b25] The Ninth Chinese Pharmacopoeia Commission of the People’s Republic of China. Pharmacopoeia of the People’s Republic of China (2010). The Medicine Science and Technology Press of China (2010).

[b26] National Meteorological Information Center, China Meteorological Administration. Chinese Meteorological data network, Beijing, China. URL: http://data.cma.cn/data/index/0b9164954813c573 (2015).

[b27] The Office for the second National Soil Survey of China. Soil Series of China. China Agricultural Press (1996).

[b28] Computer network information center, Chinese academy of sciences. Geospatial Data Cloud, Beijing, China. URL: http://www.gscloud.cn/sources/?cdataid=302&pdataid=10 (2015).

[b29] Chinese Academy of Sciences China vegetation map editor committee. Vegetation Map of the People’s Republic of China (1: 1 million). Geological Publishing House (2007).

[b30] Kira T. A newclassification of climate in easternAsia as the basis for agricultural geography. Horicultural Institute, Kyoto Univ. Kyoto. 1–23 (1945).

[b31] Xu W. D. The relation between distribution of edificator and companion in zonal vegetation and water-temperature condition in Northeast China. Acta Bot. Sin. 25, 264–274 (1983).

[b32] Guo L. P. . Key Influencing Factors on Essential Oil Component of Atractylodes Lancea and Study on its Division of Climate Adaptability. China Journal of Chinese Materia Medica. 32, 888–893 (2007). 17655137

[b33] Guo L. P., Huang L. Q., Yan H., Lv D. M. & Jiang Y. X. Habitat Characteristics for the Growth of Atractylodes Lancea Based on GIS. China J. Chin. Materia Medica. 30, 565–569 (2005). 16011274

[b34] Sun Y. Z. . Ecological Charateristics Based Productive Adaptability Zoning for Atractylodes Lancea.World Sci. & Tech. – Mode. Trad. Chin. Medicine & Materia Medica. 10, 88–92 (2008).

[b35] Sun Y. Z. . Canonical Correspondence Analysis on Distribution and Relationship of Atractylodes Lancea with their environments on Maoshani Mountain. J. TCM Uni. Hunan. 27, 218–221 (2007).

[b36] Zhang Y. . Effects of Different Microhabitas on Growth and Four Kinds of Volatile Oil Components of Atractylodes Lancea. China J. Chin. Materia Medica. 40, 4142–4148 (2015). 27071246

