# Supplementary material for: Regionalization of Chinese Material Medical Quality Based on Maximum Entropy Model: A case study of Atractylodes lancea
Source: Sci Rep. 2017 Feb 16;7:42417. doi: 10.1038/srep42417 (PMC5311955; doi:10.1038/srep42417)
Supplement: Supplementary Material 1 [file srep42417-s1.pdf]

Regionalization of Chinese Material Medical Quality Based on Maximum Entropy Model: A case study of *Atractylodes lancea*

Zhu Shoudong[1], Peng Huasheng[2], Guo Lanping[1],Xu Tongren[3],Zhang Yan[1], Chen Meilan[1],Hao Qingxiu[1], Kang Liping[1], Huang Luqi[1]*

Supplementary material 1 4 kinds of chemical composition content value of *atractylodes* at 20 sample sites

Sample No. in Sample site 1	Atractylon (mg/g)	Hinesol (mg/g)	β-eudesmol (mg/g)	Atractylodin (mg/g)
1	0.639	30.304	29.14	0.1257
2	0.952	29.756	13.832	0.2376
3	0.479	45.199	39.788	0.0852
4	0.586	34.489	31.685	0.1854
5	0.607	40.559	34.653	0.2587

Sample No. in Sample site 2	Atractylon (mg/g)	Hinesol (mg/g)	β-eudesmol (mg/g)	Atractylodin (mg/g)
1	1.025	32.434	14.551	1.597
2	0.956	15.2672	10.991	1.147
3	1.045	19.335	20.928	2.109
4	0.856	28.017	27.369	1.4138

Sample No. in Sample site 3	Atractylon (mg/g)	Hinesol (mg/g)	β-eudesmol (mg/g)	Atractylodin (mg/g)
1	1.412	31.214	21.429	0.793
2	0.993	25.76	22.364	1.088
3	1.257	28.442	22.981	0.914
4	0.741	30.472	20.391	0.965

Sample No. in Sample site 4	Atractylon (mg/g)	Hinesol (mg/g)	β-eudesmol (mg/g)	Atractylodin (mg/g)
1	0.742	19.765	23.574	1.204
2	0.573	17.684	27.652	1.836
3	1.129	16.578	22.513	0.758
4	0.875	20.367	26.783	0.774

Sample No. in Sample site 5	Atractylon (mg/g)	Hinesol (mg/g)	β-eudesmol (mg/g)	Atractylodin (mg/g)
1	0.445	29.047	20.904	1.981
2	0.347	25.318	25.341	0.758
3	0.856	22.907	21.749	1.364
4	0.245	27.354	22.004	1.172

Sample No. in Sample site 6	Atractylon (mg/g)	Hinesol (mg/g)	β-eudesmol (mg/g)	Atractylodin (mg/g)
1	0.354	25.749	25.403	0.996
2	0.834	22.983	28.962	1.35
3	0.987	26.198	25.174	0.618
4	0.471	25.106	22.353	1.125

Sample No. in Sample site 7	Atractylon (mg/g)	Hinesol (mg/g)	β-eudesmol (mg/g)	Atractylodin (mg/g)
1	0.42	25.369	26.397	1.198
2	0.39	23.184	19.321	1.201
3	0.19	15.362	22.819	1.123
4	0.202	21.723	24.037	1.807

Sample No. in Sample site 8	Atractylon (mg/g)	Hinesol (mg/g)	β-eudesmol (mg/g)	Atractylodin (mg/g)
1	1.223	26.293	26.147	1.373
2	1.126	27.724	22.391	1.42

3	1.452	29.047	25.517	1.3495
4	1.117	29.155	24.887	1.326

Sample No. in Sample site 9	Atractylon (mg/g)	Hinesol (mg/g)	β-eudesmol (mg/g)	Atractylodin (mg/g)
1	1.552	28.145	16.552	7.07
2	2.086	35.539	23.086	6.933
3	4.293	45.384	42.293	7.185

Sample No. in Sample site 10	Atractylon (mg/g)	Hinesol (mg/g)	β-eudesmol (mg/g)	Atractylodin (mg/g)
1	1.258	37.143	58.754	0
2	2.069	42.876	48.517	0
3	1.685	9.823	50.937	0

Sample No. in Sample site 11	Atractylon (mg/g)	Hinesol (mg/g)	β-eudesmol (mg/g)	Atractylodin (mg/g)
1	7.564	13.975	19.317	6.045
2	8.194	10.658	14.568	12.435
3	6.284	9.115	10.513	5.699
4	7.654	14.495	9.295	7.758

Sample No. in Sample site 12	Atractylon (mg/g)	Hinesol (mg/g)	β-eudesmol (mg/g)	Atractylodin (mg/g)
1	11.399	9.865	9.613	5.531
2	12.034	8.412	8.512	4.904
3	12.577	8.336	9.661	4.995
4	12.205	7.404	9.03	5.218

Sample No. in Sample site 13	Atractylon (mg/g)	Hinesol (mg/g)	β-eudesmol (mg/g)	Atractylodin (mg/g)
1	0.684	13.645	7.056	8.026
2	1.102	15.712	7.631	6.339

3	0.963	15.336	6.195	5.078
4	0.994	14.803	6.382	7.584

Sample No. in Sample site 14	Atractylon (mg/g)	Hinesol (mg/g)	β-eudesmol (mg/g)	Atractylodin (mg/g)
1	11.043	2.054	2.145	3.099
2	11.632	1.971	1.991	2.417
3	9.541	2.409	1.573	2.553
4	10.897	1.75	2.449	3.108

Sample No. in Sample site 15	Atractylon (mg/g)	Hinesol (mg/g)	β-eudesmol (mg/g)	Atractylodin (mg/g)
1	9.145	3.845	3.874	2.011
2	9.772	4.023	3.745	1.964
3	12.543	4.775	3.556	1.883
4	8.364	4.546	4.193	2.067

Sample No. in Sample site 16	Atractylon (mg/g)	Hinesol (mg/g)	β-eudesmol (mg/g)	Atractylodin (mg/g)
1	6.087	6.987	7.258	6.301
2	7.158	6.425	6.998	9.336

Sample No. in Sample site 17	Atractylon (mg/g)	Hinesol (mg/g)	β-eudesmol (mg/g)	Atractylodin (mg/g)
1	3.205	9.872	3.698	6.074
2	3.158	10.963	4.741	7.148
3	2.874	10.521	4.087	6.91
4	3.698	9.541	5.674	6.218

Sample No. in Sample site 18	Atractylon (mg/g)	Hinesol (mg/g)	β-eudesmol (mg/g)	Atractylodin (mg/g)
1	2.541	8.204	3.552	5.987
2	2.604	9.331	4.258	6.024

3	1.993	10.826	4.233	4.361
4	2.806	9.395	5.841	5.776

Sample No. in Sample site 19	Atractylon (mg/g)	Hinesol (mg/g)	β-eudesmol (mg/g)	Atractylodin (mg/g)
1	1.231	9.337	3.958	5.376
2	2.415	10.325	9.136	5.687
3	1.526	11.254	6.147	4.873
4	2.085	9.451	4.84	5.994

Sample No. in Sample site 20	Atractylon (mg/g)	Hinesol (mg/g)	β-eudesmol (mg/g)	Atractylodin (mg/g)
1	2.087	18.383	16.924	3.497
2	1.78	17.552	16.418	4.052
3	1.806	19.364	14.511	3.554
4	2.364	16.385	14.76	3.669
